# Modified Technique for CAD/CAM Guided Implant Planning in the Presence of Existing Hopeless Teeth

**DOI:** 10.30476/DENTJODS.2019.77838

**Published:** 2020-03

**Authors:** Andrew C. Johnson, Vinay Jain, Swati Ahuja

**Affiliations:** 1 Dept. of Prosthodontics, University of Tennessee Health Science Center, College of Dentistry, Memphis, Tennessee, USA

**Keywords:** CAD/CAM, Implant planning, Prosthetically driven, Surgical guide

## Abstract

Immediate placement of dental implants presents many challenges, especially when partial or complete fixed restorations are the intended prosthetic outcome. With modern advancements in CAD/CAM technology, the ease and predictability of such complex cases is vastly improved. However, certain clinical situations remain that preclude the traditional implementation of this controlled approach to implant planning/placement and the current solutions to these problems each impose some level of compromise. This article describes a technique permitting both prosthetically-driven implant planning and increased surgical guide accuracy in situations where existing hopeless teeth would otherwise impede optimal treatment.

## Introduction

The combined utility of cone beam computed tomography (CBCT), virtual dental implant planning computer software, and rapid prototyping of surgical guides has dramatically improved the accuracy of dental implant placement and the quality of the associated prostheses [ 1 - 7 ]. These technologies provide complete three-dimensional visualization not only of patient anatomy but also the proposed prosthetic contours thereby facilitating prosthetically-driven implant placement with an excellent control [ 8 - 12 ]. These methods are optimally suited for planning implant placement in both edentulous and partially dentate cases in which the existing natural teeth are to be maintained. In situations where hopeless teeth remain, it is recommended that the teeth be extracted prior to scanning/planning of implants [ 13 - 14 ]. While eliminating hopeless teeth (prior to radiographic template construction) prevents their obscuring both the visible osseous architecture as well as the proposed restorative contours, it does require additional surgical and healing phases. Pre-emptive extraction also discards any contributions those teeth may provide in surgical guide fixation leaving the accuracy of implant placement entirely at the mercy of less stable soft tissue [ 6 ]. Cantoni et al. [ 15 ] described a technique for developing an implant plan around existing hopeless teeth using a two-piece scanning template and a dual scan approach [ 15 ]. However, the hopeless teeth do not serve as positioning indices for the surgical guide if all of the remaining teeth are extracted in the course of implant surgery. Moreover, this technique does not permit alteration of the diagnostic tooth setup without added laboratory procedures [ 15 ].

The technique presented in this article offers another solution for the clinical situations where hopeless teeth are to be maintained up to the time of implant placement allowing their utilization in orienting and fixating the radiographic and surgical templates.

## Technique

**Figure1:**
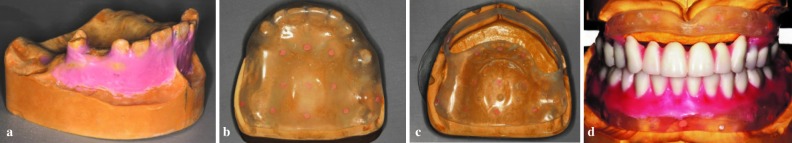
**a:** Undercuts blocked using modeling wax, **b:** Fiduciary markers added on the palatal surface and along the border extensions of the trial denture base, **c:** Radiographic template placed on the adjusted maxillary cast, **d:** Maxillary prosthetic teeth waxed on the radiograph-ic template to achieve desired esthetics and occlusion

**Figure2:**
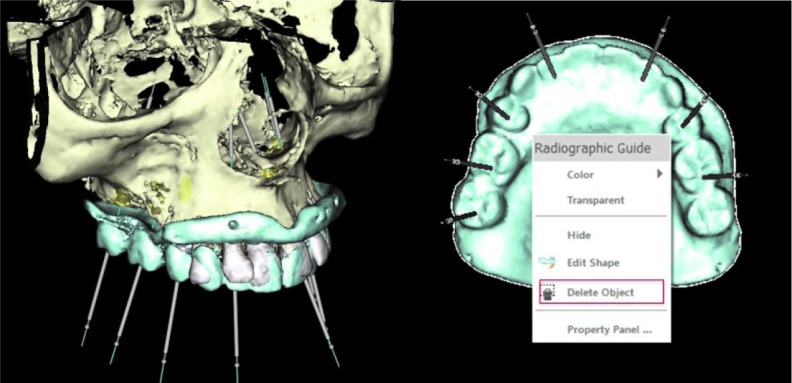
**a:** Implants planned in the planning software by merging the patient scan with the scan of the modified radiographic template, **b:** Mod-ified radiographic template deleted following implant planning

**Figure3:**
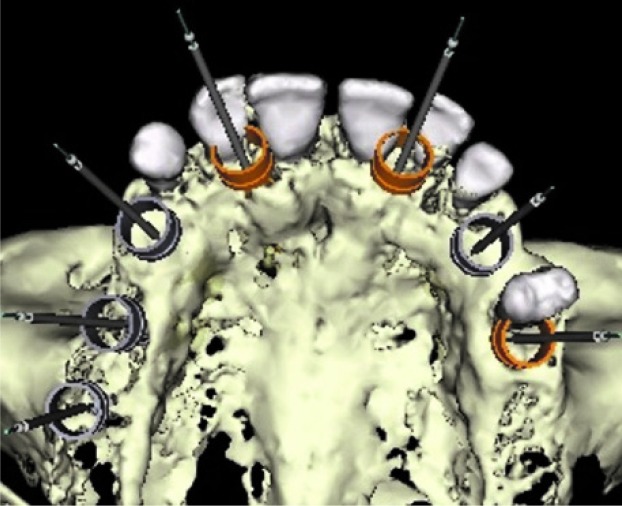
Implants and guide sleeve positions can be visualized relative to the existing teeth

**Figure4:**
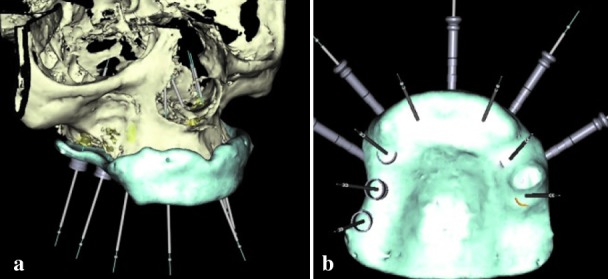
**a:** Implants originally planned on modified radiographic template can now be visualized relative to the contours of the original radio-graphic template, **b:** Anchors pins added to implant planning

**Figure5:**
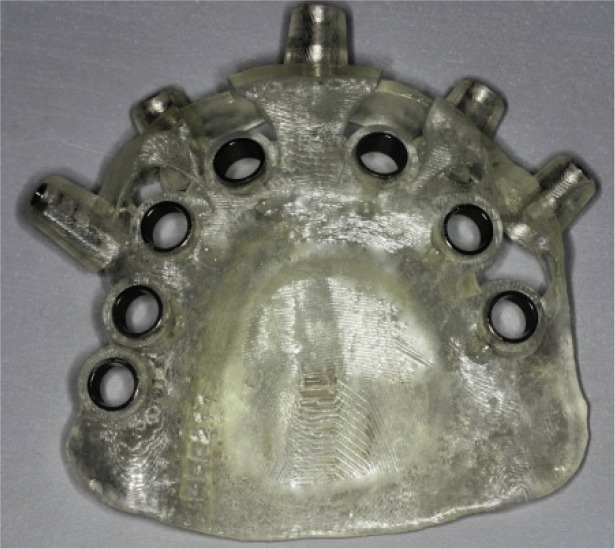
Stereolithic surgical guide

1. Make maxillary and mandibular diagnostic impressions with alginate (Jeltrate, Dentsply Caulk) in stock trays. Mount the casts on a semi-adjustable articulator (Whipmix 2240, WhipMix Corp) using the facebow and interocclusal records.
2. Block out all undercuts (Figure 1a) and fabricate maxillary acrylic trial denture base (Ortho Resin; GC America Inc., Alsip, Ill) covering the existing hopeless teeth and extended up to the depth of the vestibule.
3. Add radiopaque fiducial markers on the palatal surface and along the border extensions of the trial denture base such that they do not interfere with future prosthetic teeth arrangement. This trial denture base can now serve as radiographic template (RT) (Figure 1b) so that they do not interfere with future prosthetic teeth arrangement.
4. Try the RT in the patient’s mouth and adjust it to ensure optimal fit. Register an interocclusal record with the RT in the mouth using a bite registration material (Regisil, Dentsply Caulk).
5. Acquire a CBCT (Kodak 9000, Carestream) scan of the patient (P-I) with the RT and the interocclusal record placed in the oral cavity and a second CBCT scan (T-I) of the RT by itself.
6. Examine the existing maxillary anterior teeth clinically: the midline, anterior occlusal plane, gingival line, smile line and the labio-lingual position of the teeth. Assess and record the desired changes in the maxillary anterior tooth positions to achieve optimal esthetics.
7. Make keyways on the land area of the cast and fabricate a vinyl polysiloxane putty (Aquasil putty, Dentsply Caulk) index to record the position of the existing maxillary anterior teeth. Eliminate all the maxillary hopeless teeth on the cast and adjust the residual ridge contours, as necessary. Place the RT on the adjusted maxillary cast (Figure 1c), adjust the RT and wax the maxillary prosthetic teeth on the RT using the putty index and clinical records to achieve the desired esthetics and occlusion (Figure 1d). Areas bearing the fiducial markers must be left unaltered. Acquire a new CBCT scan (T-II) of the modified RT (MRT)/ RT (with all the prosthetic teeth waxed to it) by itself.
8. Using the implant planning software (Nobel Clinician, Nobel Biocare) combine the patient scan (P-I) and the scan of the modified RT (T-II), via fiducial marker correlation.
9. Plan the implants optimally by visualizing the anatomic structures and the final position of prosthetic teeth as desired in the definitive prosthesis. Once the implant plan is finalized (Figure 2a), it is then necessary to incorporate this plan within the con tours of the unmodified RT. To accomplish this, the MRT is selected in the software with the cursor and then deleted (Figure 2b). The implants and guide sleeve positions can now be visualized relative to the existing teeth (Figure 3).
10. Adding a new radiographic guide option in the implant planning software allows the RT scan (T-I) to be merged with the patient scan (P-I). The originally planned implants can now be visualized relative to the contours of the original RT (Figure 4a). The anchor pins are planned (Figure 4b), the surgical template is finalized in the software and the data is sent to the manufacturing facility for its fabrication (Figure 5).

## Summary

This technique affords patients and providers the option of retaining their hopeless teeth throughout the entire pre-implant treatment phase. It not only aids in utilizing the existing teeth as surgical guide positioning indices but also permits clinician-directed prosthetic tooth positioning. Using modified radiographic guide for implant planning permits visualization of the definitive prosthesis contours and final positions of prosthetic teeth during virtual implant planning. Because this method precludes the need for a fully developed scanning template, it allows for much earlier acquisition of a CBCT scan for tentative surgical diagnosis as well as eventual implant planning and surgical template fabrication. 

This technique can thus minimize radiation exposure to the patient (since only one patient scan is required) while both optimizing and expediting implant treatment planning. 
